# Nutritional Heterogeneity Among *Aspergillus fumigatus* Strains Has Consequences for Virulence in a Strain- and Host-Dependent Manner

**DOI:** 10.3389/fmicb.2019.00854

**Published:** 2019-04-24

**Authors:** Laure Nicolas Annick Ries, Jacob L. Steenwyk, Patrícia Alves de Castro, Pollyne Borborema Almeida de Lima, Fausto Almeida, Leandro José de Assis, Adriana Oliveira Manfiolli, Azusa Takahashi-Nakaguchi, Yoko Kusuya, Daisuke Hagiwara, Hiroki Takahashi, Xi Wang, Joshua J. Obar, Antonis Rokas, Gustavo H. Goldman

**Affiliations:** ^1^Faculdade de Medicina de Ribeirão Preto, Universidade de São Paulo, São Paulo, Brazil; ^2^Department of Biological Sciences, Vanderbilt University, Nashville, TN, United States; ^3^Faculdade de Ciências Farmacêuticas de Ribeirão Preto, Universidade de São Paulo, São Paulo, Brazil; ^4^Medical Mycology Research Center, Chiba University, Chiba, Japan; ^5^Department of Microbiology and Immunology, Dartmouth Geisel School of Medicine, Lebanon, NH, United States

**Keywords:** *Aspergillus fumigatus*, clinical isolates, carbon and nitrogen catabolite repression, genome comparison, cell wall, virulence

## Abstract

Acquisition and subsequent metabolism of different carbon and nitrogen sources have been shown to play an important role in virulence attributes of the fungal pathogen *Aspergillus fumigatus*, such as the secretion of host tissue-damaging proteases and fungal cell wall integrity. We examined the relationship between the metabolic processes of carbon catabolite repression (CCR), nitrogen catabolite repression (NCR) and virulence in a variety of *A. fumigatus* clinical isolates. A considerable amount of heterogeneity with respect to the degree of CCR and NCR was observed and a positive correlation between NCR and virulence in a neutropenic mouse model of pulmonary aspergillosis (PA) was found. Isolate Afs35 was selected for further analysis and compared to the reference strain A1163, with both strains presenting the same degree of virulence in a neutropenic mouse model of PA. Afs35 metabolome analysis in physiological-relevant carbon sources indicated an accumulation of intracellular sugars that also serve as cell wall polysaccharide precursors. Genome analysis showed an accumulation of missense substitutions in the regulator of protease secretion and in genes encoding enzymes required for cell wall sugar metabolism. Based on these results, the virulence of strains Afs35 and A1163 was assessed in a triamcinolone murine model of PA and found to be significantly different, confirming the known importance of using different mouse models to assess strain-specific pathogenicity. These results highlight the importance of nitrogen metabolism for virulence and provide a detailed example of the heterogeneity that exists between *A. fumigatus* isolates with consequences for virulence in a strain-specific and host-dependent manner.

## Introduction

Opportunistic fungal infections have become a major concern for global population health, as they are predicted to lead to more deaths annually than malaria and tuberculosis (Denning and Bromley, 2015). Of the few hundred identified species in the *Aspergillus* genus (Visagie et al., 2014), *Aspergillus fumigatus* is the primary and most common causative agent of opportunistic infectious disease in humans (Dagenais and Keller, 2009). Within the species of *A. fumigatus*, SNPs (single nucleotide polymorphisms) and InDels (insertion–deletions) confer great heterogeneity to isolates that is predicted to have implications for virulence traits and pathogenicity altogether (Keller, 2017). In immunocompetent hosts, *A. fumigatus* rarely causes infection except if a pre-existing condition is present (Norton and Kobusingye, 2013). In immunocompromised patients, however, *A. fumigatus* can cause aspergillosis, a term collectively used for a spectrum of mycological diseases caused by species in the genus *Aspergillus* whose severity depends on the underlying disturbance in the immune system, induced by either chemical treatment, radiotherapy, immunodeficiency diseases or genetic disorders (Abad et al., 2010).

*Aspergillus fumigatus* pathogenicity is a multifactorial trait, encompassing a series of survival and fitness-enhancing factors, that determine virulence. Nutrient acquisition and subsequent metabolic processes are crucial for initial host colonization and promote invasion as well as long-term survival within the host. Micro-nutrients such as iron, zinc and copper are required in small amounts but are essential for establishing and maintaining infection (Schrettl and Haas, 2011; Baldin et al., 2015; Vicentefranqueira et al., 2015; Wiemann et al., 2017). In addition, carbon and nitrogen compounds must be acquired in large quantities as they sustain cellular biosynthetic processes (Ramachandra et al., 2014), although their utilization and role during infection have been less well studied than micronutrient acquisition. The importance of carbon and nitrogen utilization during infection is largely based on transcriptional studies where *A. fumigatus* has been exposed to immune cells *in vitro* (Sugui et al., 2008) or on studies where genes encoding enzymes of central carbon and nitrogen metabolism have been deleted, resulting in strains with attenuated virulence in a murine model of invasive aspergillosis [for extensive reviews please refer to Krappmann and Braus (2005), Rhodes (2006), Willger et al. (2009), Beattie et al. (2017), and Ries et al. (2018a)]. Nevertheless, carbon and nitrogen primary metabolic features are important for *A. fumigatus* pathogenesis, as was shown recently for the transcriptional mediator of carbon catabolite repression (CCR) CreA, that is required for growth and disease progression in a murine model of invasive pulmonary aspergillosis (IPA) (Beattie et al., 2017). CCR is a metabolic strategy by which the fungus selects the energetically most favorable carbon source, such as glucose, thereby providing rapid energy for growth and niche colonization (Ruijter and Visser, 1997). Similarly, nitrogen catabolite repression (NCR) favors the utilization of ammonium and glutamine because they are easily assimilated and can readily be used for protein synthesis (Wong et al., 2008).

In addition to sustaining growth and biosynthetic cellular processes, carbon and nitrogen metabolism also affect fungal traits such as enzyme secretion and cell wall integrity, both of which are essential pathogenicity determinants (Andersen, 2014). Proteases are a group of enzymes that are predicted to play an important role during infection as they can degrade host lung tissue and subsequently promote localized invasion and colonization (Kogan et al., 2004; Mavor et al., 2005; Namvar et al., 2015). The genome of *A. fumigatus* encodes an array of proteases that belong to different classes and whose secretion is regulated in a substrate-dependent manner (Farnell et al., 2012). The cell wall is essential for fungal survival, as it is the main line of defense and site of interaction between the fungus and host cells (Abad et al., 2010). The cell wall makes up around one-quarter of the total fungal biomass and is composed of proteins and a complex intertwined network of polysaccharides such as glucans, chitin and galactomannans (Gastebois and Aimanianda, 2009). Both protease secretion and the cell wall have been determined as primary virulence factors, with the former playing an important role in soliciting a pro-inflammatory response during bronchopulmonary allergies (Farnell et al., 2012), and with the latter having both immune-evasive and –modulatory properties (Abad et al., 2010; Chai et al., 2011; Cramer et al., 2011; Briard et al., 2016).

The aim of this study was to investigate CCR and NCR with respect to protease secretion in several *A. fumigatus* clinical isolates to determine if a relationship between both these metabolic processes and virulence exists. Although a considerable amount of heterogeneity exists in all tested clinical isolates with respect to the degree of CCR and NCR, a positive correlation between NCR, but not CCR, and virulence in a neutropenic mouse model of IA was found. Strain Afs35 was selected for further metabolic and genomic analysis, as it secreted high amounts of proteases under different NCR conditions. Metabolome analysis in physiologically relevant carbon sources indicated an accumulation of intracellular sugars that serve as cell wall polysaccharide precursors in strain Afs35 when compared to strain A1163. Furthermore, Afs35 genome analysis showed an accumulation of missense substitutions in *prtT*, encoding the main the regulator of protease gene transcription, and in genes encoding enzymes involved in cell wall chitin and glucan metabolism. Substantial differences in the quantities of conidial cell wall enzymes were confirmed by mass spectrometry between both strains. Although no significant difference in virulence between strains A1163 and Afs35 was found in a neutropenic mouse model, strain Afs35 was attenuated in virulence in a triamcinolone murine model of pulmonary aspergillosis (PA). Together, the aforementioned results strongly suggest that *A. fumigatus* genetic diversity is tightly linked to strain-specific virulence traits and the type of host immune suppression.

## Materials and Methods

### *A. fumigatus* Strains Used

All *A. fumigatus* strains used in this study were clinically isolated from both immunocompromised male and female patients that suffered from different types of cancer or other, unknown diseases (Table 1). All strains have previously been sequenced and confirmed to be *A. fumigatus* (Table 1). All strains were obtained from patients who are above 18-years old by written informed consent. People with disabilities or endangered animal species were not used in our studies.

**Table 1:** Names and origin of clinical isolates used in this study.

Sample origin	Sample/strain name	Sex/age	Pathology	Date isolated	Patient treated with echinocandins and/or azoles	References
BL	MO89263	F/70	Digestive system cancer	28/01/2014	YES	Lind et al., 2017
BS	MO91298	F/70	Digestive system cancer	03/03/2014	YES	Lind et al., 2017
S	MO54056	F/73	Chronic lymphoid leukemia	11/04/2011	YES	Lind et al., 2017
S	MO79587	F/73	Chronic lymphoid leukemia	19/07/2013	YES	Lind et al., 2017
S	MO69250	F/54	Chronic lymphoid leukemia	09/01/2013	YES	Lind et al., 2017
S	MO78722	F/54	Chronic lymphoid leukemia	04/07/2013	YES	Lind et al., 2017
S	MO68507	M/44	Chronic myeloid leukemia	20/12/2012	YES	Lind et al., 2017
–	A1163/CEA10	–	Invasive pulmonary aspergillosis (IPA)	–	–	Fedorova et al., 2008
–	ATCC 13073	–	–	–	–	www.atcc.org
BL	ASFU 1643	–	–	18/05/2011	YES	
–	R21	–	–	–	–	Niki et al., 1991
–	Afs35	–	–	–	–	Krappmann et al., 2006
LB	Af293	–	IPA	1993	–	http://www.westerdijkinstitute.nl/


*BL, bronchial lavage; BS, bronchial secretion; S, sputum; LB, lung biopsy; –, not applicable, unknown.*

### Growth Media and Conditions

All experiments on all strains were carried out at 37°C on minimal medium (MM) supplemented with different carbon sources as described previously (Ries et al., 2016). Reagents were obtained from Sigma Aldrich (Darmstadt, Germany) unless otherwise stated. Where required, MM was supplemented with the indicated concentrations of 2-deoxyglucose (2DG), allyl alcohol (AA), congo red (CR), calcofluor white (CFW) or caspofungin (CF). MM without any nitrogen source (AMM) was prepared in the same way as conventional MM, except that all nitrogen compounds were taken from the 20× salt solution and the 5× trace element solution (for detailed recipes, refer to Ries et al., 2016). A 10% w/v solution of dry-skimmed milk was prepared separately and added to MM or AMM to a final concentration of 1% v/v. Where required, AMM was supplemented with 0.03% w/v urea or 10 mM ammonium tartrate. Heat maps were generated using the program MEV 4.9.0 and are based on the strain-specific average growth or protease secretion index in each specified condition.

Plates were inoculated with 10^5^ spores per strain. All serial dilutions started at 10^5^ spores before 1 in 10 dilutions were made to generate 10^4^, 10^3^, and 10^2^ strain spore suspensions. Strains were left to grow for either 48 h or 120 h. To determine the protease secretion index and ensure strain-specific normalization, the diameter for the protease halo and the colony was measured and divided by the diameter of the colony only.

### Alcohol Dehydrogenase Activity

Alcohol dehydrogenase activity in whole cell protein extracts of biological triplicates was determined as described previously (Ries et al., 2018b). Briefly, total protein was extracted from mycelia grown in the specified conditions and protein concentration was determined by Bradford assay (Bio-Rad, Hercules, CA, United States) according to manufacturer’s instructions. In a 96-well, transparent, flat-bottom plate, 5 μg of total protein extract was mixed with buffer and NAD^+^ to a final volume of 180 μl and incubated at 37°C for 10 min. The reaction was started by adding 20 μl of a 10 M ethanol solution and absorbance was read at 340 nm for 10 min at 37°C.

### Metabolomics

Metabolite extraction, derivatization, identification and quantification was carried out as described previously (Ries et al., 2018b) on five biological replicates. Briefly, metabolites were extracted from ∼ 5 mg of dry-frozen mycelia with 1 ml of MTBE (methyl tert-butyl ether): methanol:water in a 3:1:1 (v/v/v) ratio. Drying and derivatization were carried out on 100 μl of the extracted polar phase, before 1 μl of the derivatized sample was analyzed by gas chromatography and mass spectrometry. Samples were normalized by dry weight.

### Conidia Cell Wall Protein Extraction, Identification and Quantification by Mass Spectrometry

Total proteins from the fungal conidial surface were extracted as described previously (Beauvais et al., 2013; Leite et al., 2015) and quantified as follows. Protein concentration was determined using the BCA Protein Assay Kit (Thermo Scientific, Waltham, MA, United States) according to the manufacturer’s instructions. Samples were dried and applied on to the NanoLC PROXEON EASY-nLC II coupled with LTQ Orbitrap Velos ETD mass spectrometer (Thermo Scientific). The acquired data were automatically processed using Mascot Daemon. The identified peptides were grouped into proteins using the Proteome Software Scaffold Q+S, and a list of identified proteins was established using an error lower than 5%. The data were compared with *A. fumigatus* proteomes (Af293 or A1163) at http://www.aspgd.org.

### *In vitro* Macrophage Assays and Cytokine Quantification

All experiments were performed with murine bone marrow-derived macrophages (BMDM). BMDM preparation, phagocytosis and killing assays were performed exactly as described in Rocha et al. (2018).

Cytokine levels of TNF-α, IL-12p40, and IL-1β in the supernatants of BMDM challenged with fungal spores were quantified as described previously with the following modifications. All cytokine levels were measured by capture enzyme-linked immunosorbent assay (ELISA) using BD Biosciences (San Diego, CA, United States) antibodies, according to manufacturer’s instructions. Cytokine concentrations were determined as a reference to standard curves, which were established using murine recombinant cytokines.

Quantitative real-time PCR (qRT-PCR) was performed as previously described (Almeida et al., 2017), with modifications. BMDMs were stimulated for 6 h with fungal spores before macrophage RNA was extracted using Trizol Reagent (Life Technologies, Camarillo, CA, United States), according to manufacturer’s instructions. cDNA was prepared from RNA using oligo(dT) and the ImProm-II Reverse Transcription System (Promega, Fitchburg, WI, United States), according to manufacturer’s instructions. All qRT-PCR reactions were performed in a total volume of 15 μl, containing SsoFast^TM^ EvaGreen Supermix (Bio-Rad) and using the Bio-Rad CFX96 Real-Time PCR System. PCR conditions were as follows: 50°C for 2 min, 95°C for 10 min, and 40 cycles of 95°C for 15 s and 60°C for 1 min using the following primers: β-actin (F: 5′-AGCTGCGTTTTACACCC-3′/R: 5′-AAGCCATGCCAATGTTGTCT-3′); T-bet (F: CACTAAGCAAGGACGGCGAA/R: CCACCAAGACCACATCCAC); GATA-3 (F: AAGAAAGGCATGAAGGACGC/R: GTGTGCCCA TTTGGACA TCA); ROR-γ t (F: TGGAAGATGTGGACTTCGTT/R: TGGTTCCCCAAGTTCAGGAT). All transcript levels were quantified using the ΔΔCt method and normalized relative to β-actin gene expression.

### *In vivo* Neutropenic and Corticosteroid Murine Infections

Induction of neutropenia in inbred (using 10 mice/fungal strain and 5 animals for the negative control) BALB/c female mice (University of São Paulo), *A. fumigatus* spore suspension preparations and murine infection by nasal instillation was carried out exactly as described previously (Manfiolli et al., 2017). Briefly, neutropenia was induced by intraperitoneal administration of cyclophosphamide (150 mg/kg of body weight) on days -4, -1, and 2 prior to and post infection and by subcutaneous injection of hydrocortisone acetate (200 mg/kg body weight) on day -3. *A*. *fumigatus* conidia were harvested in PBS, filtered, washed, counted and re-suspended at a concentration of 5.0 × 10^6^ conidia/ml before conidia viability was tested as described previously (Manfiolli et al., 2017). Mice were anesthetized by halothane inhalation and infected with 1.0 × 10^5^ conidia in 20 μl of PBS.

For the corticosteroid-induced murine model of invasive aspergillosis, outbred (using 15 mice/fungal strain and 10 animals for the negative control) female CD-1 mice (Strain Code: 022) were purchased from Charles River Laboratories. All mice were 7–10 weeks of age at the time of challenge. *A. fumigatus* strains were grown on glucose minimal media (GMM) agar plates for 3 days at 37°C. Conidia were harvested by adding 0.01% Tween 80 to plates and gently scraping conidia from the plates using a cell scraper. Conidia were then filtered through sterile Miracloth, were washed and re-suspended in phosphate buffered saline (PBS), and counted on a hemocytometer. CD-1 mice were treated with 40 mg/kg Kenalog-10 (Bristol-Myers Squibb, New York, NY, United States) by subcutaneous injection 24 h prior to fungal inoculation under isoflurane anesthesia. Corticosteroid-treated mice were challenged with 2 × 10^6^ conidia. Conidia were prepared in 100 μl sterile PBS and administered to mice intratracheally while under isoflurane anesthesia. Mock challenged mice were given 100 μl sterile PBS. Mice were monitored at least three times a day for signs of disease for 14 days post-inoculation. Survival was plotted on Kaplan–Meier curves, and statistical significance between curves was determined using the Mantel–Cox log rank. For end-point analysis, mice were euthanized using a lethal overdose of pentobarbital at the indicated time after *A. fumigatus* challenge.

### Bronchoalveolar Lavage Fluid (BALF) Preparation

Bronchoalveolar lavage fluid (BALF) was collected 72 h after infection by washing the lungs with 2 ml of PBS containing 0.05 M EDTA. BALF was clarified by centrifugation and stored at -20°C until analysis. After centrifugation, the cellular component of the BAL was re-suspended in 200 μl of PBS and total BAL cells were determined by hemocytometer count. BAL cells were subsequently spun onto glass slides using a Cytospin4 cytocentrifuge (Thermo Scientific) and stained with Diff-Quik stain set (Siemens, Munich, Germany) for differential counting.

### Quantification of Lung Damage and Leakage

To assess lung damage, BALF from 72 h post-inoculation was analyzed by measuring lactate dehydrogenase levels using a CytoTox 96^®^ Cytotoxicity Assay (Promega) following the manufacturer’s instructions. To assess vascular/pulmonary leakage, BALF was analyzed using an Albumin BCG Reagent Set (Eagle Diagnostics, Cedar Hill, TX, United States). A standard curve was made by diluting the calibrator in PBS/EDTA. Then 100 μl of sample or standard was transferred to a 96 well flat-bottomed plate, mixed with 100 μl of BCG reagent, let sit at RT (room temperature) for 5 min and then read on a plate reader at 630 nm.

### Luminex Assay for Cytokine and Chemokine Secretion

Bronchoalveolar lavage fluid from mice challenged with each *A. fumigatus* strain was collected at 72 h post-inoculation. Samples were analyzed for cytokines and chemokines using a custom ProCarta Mouse Cytokine & Chemokine 22-plex (Life Technologies). Plates were read using a BioPlex 200 (Bio-Rad) in the Immune Monitoring and Flow Cytometry Core Facility at Dartmouth College.

### Statistical Analysis

Statistical significance was determined by a Mann–Whitney *U* test, one-way ANOVA using a Bonferroni post-test, or Kruskal–Wallis one-way ANOVA with Dunn’s post-test through the GraphPad Prism 5 software as outlined in the figure legends.

### Ethics Statement

The principles that guide our studies are based on the Declaration of Animal Rights ratified by the UNESCO on January 27, 1978, in its eighth and 14th articles. All protocols used in this study were approved by the local ethics committee for animal experiments from the Campus of Ribeirão Preto, Universidade de São Paulo (Permit Number: 08.1.1277.53.6; Studies on the interaction of *A. fumigatus* with animals) or the Dartmouth College Institutional Animal Care and Use Committee (Protocol Number: 00002168). All corticosteroid-induced invasive aspergillosis animal experiments were approved by the Dartmouth College Institutional Animal Care and Use Committee. All animals were housed in groups of five within individually ventilated cages and were cared for in strict accordance with the principles outlined by the Brazilian College of Animal Experimentation (Princípios Éticos na Experimentação Animal—Colégio Brasileiro de Experimentacão Animal, COBEA) and Guiding Principles for Research Involving Animals and Human Beings, American Physiological Society. All efforts were made to minimize suffering. Animals were clinically monitored at least twice daily and humanely sacrificed if moribund (defined by lethargy, dyspnoea, hypothermia, and weight loss). All stressed animals were sacrificed by cervical dislocation.

### Whole Genome Sequencing (WGS)

Whole genome sequencing (WGS) of strain Afs35 was carried out as described previously (Takahashi-Nakaguchi et al., 2015) with the following modifications. Genomic DNA (gDNA) was extracted using the phenol-chloroform method from mycelia grown in Potato dextrose broth (PDB) (Thermo Scientific) for 18 h at 37°C. The gDNA was fragmented with the Covaris S2 (Covaris, Woburn, MA, United States) sonicator in a volume of 130 μL to generate 500 bp fragments (Duty factor [%]:5, Peak incidence power [W]:105, Cycles per burst:200, Time [s]:80) according to the manufacturer’s instructions. DNA libraries were prepared using the NEBNext Ultra DNA Library Prep Kit for Illumina (New England Biolabs NEB, Ipswich, MA, United States) according to the manufacturer’s instructions. Library quality was assessed using Agilent 2100 Bioanalyzer (Agilent Technologies, Santa Clara, CA, United States) and 100 bp paired-end sequencing was carried out with HiSeq1500 (Illumina, San Diego, CA, United States) according to manufacturer’s instructions. The raw sequencing data is available in the DDBJ (DNA Data Bank of Japan) Short Read Archive under accession number DRA007135.

### Afs35 Genome Quality Control and Read Mapping

To identify mutations between *A. fumigatus* strain Afs35 and A1163, strict quality control of sequence reads and read mapping were performed as previously described (Steenwyk and Rokas, 2017). Briefly, Trimmomatic, version 0.36 (Bolger et al., 2014) was used to remove low quality base pairs with the following parameters: leading:10, trailing:10, sliding window:4:20, and minlen:50. Next, trimmed Afs35 reads were mapped to the strain A1163 genome using Bowtie2, version 2.3.4 (Langmead and Salzberg, 2012), using the “–sensitive” parameter. The resulting Table of mapped reads was converted to bam format and indexed using the “-b” and “-u” parameters as arguments along with the Samtools, version 1.3.1 (Li et al., 2009), “view” function. The resulting Table was then sorted using the Samtools “sort” function.

### Afs35 Genome Mutation Identification and Effect Prediction

To identify single nucleotide polymorphisms (SNPs) and insertions and deletions (indels) in the genome of Afs35 when compared to the genome of A1163, a mpileup Table was created which details information about reads at each base-pair position. To do so, the resulting bam Table from previous steps was used as input to Samtools with the “mpileup” function. The resulting mpileup Table was used as input to VarScan, version 2.3.9 (Koboldt et al., 2012), with the “mpileup2snp” and “mpileup2indel” functions to identify SNPs and indels, respectively. For identified SNPs and indels, a minimum average base quality score of 20, a minimum average coverage of 15, a minimum variant allele frequency of 1.00 and a *p*-value threshold of 0.01 according to Fischer’s Exact test (Gibbons, 2014) were required. SNPs and indels were annotated (e.g., synonymous, non-synonymous, frameshift, etc.) and the outcome and degree of effect of each mutational event (e.g., high, moderate, or low impact on gene function) was predicted using SnpEff, version 4.3t (Cingolani et al., 2012) and a custom created database based on the A1163 genome.

To identify and quantify copy number variants (CNVs) in the Afs35 genome, a read-depth based approach was implemented. In brief, read-depth based approaches summarize read-depth information in predefined genomic bins, correct for GC sequencing biases, and then identify statistically significant deviations in over- or under-representation in read-depth to identify putatively duplicated and deleted loci, respectively. To do so, CNV loci were first identified using two programs: (i) Control-FREEC, version 9.1 (Boeva et al., 2011, 2012) and (ii) CNVnator, version 0.3.2 (Abyzov et al., 2011). These programs were chosen because they have low false positive rates and high true positive rates (Abyzov et al., 2011; Duan et al., 2013). Specific settings for Control-FREEC include a window size and step of 1,000, a “breakPointType” parameter set to 2, a “telocentromeric” value of 10,000, and a minimum and maximum expected GC value of 0.3 and 0.5, respectively. CNVnator was used with default settings. Loci that were significantly determined to be copy number variable were identified using a Wilcoxon Rank Sum test (Wallace, 2004) and a Kolmogorov–Smirnov test (Panchenko, 2006) using a script provided by the developers of Control-FREEC^1^ or a *T*-test (Ugoni and Walker, 1995) as provided in the CNVnator output. For each test, a *p*-value cut-off of 0.01 was used. Next, the results from each program were integrated such that only CNV regions identified by both programs as duplications or deletions were maintained. Among duplicated CNV regions, the predicted values between the two programs were averaged and rounded to the nearest whole number (Abyzov et al., 2011). Next, SNPs that overlapped with loci predicted to be deleted in Afs35 were removed because these SNPs were likely inferred from reads that were erroneously mapped in neighborhoods of no-to-low sequencing depth.

To calculate the divergence between the genomes of Afs35 and A1163, the number of base pairs affected by any type of mutation was divided by the genome assembly size.

### Statistical Assessment of the Relationship Between Growth Phenotype and Mouse Survival

To determine if growth inhibition and protease secretion of 9 *A. fumigatus* strains in the presence of CCR and NCR conditions (totalling 19 different conditions/media) was correlated with mouse survival, the Spearman rank correlation coefficient (SRCC) and multiple linear regression analysis in R, version 3.3.2 (R Development Core Team, 2008) was conducted. The SRCC was calculated as described^2^. The data around the mean for each of the 19 conditions was first transformed using the “scale()” function with the “center” parameter set to TRUE. These data were then used to build linear models to assess the correlation between mouse survival and growth phenotypes. However, since multiple linear regression analysis requires fewer dependent variables (i.e., 19 growth phenotypes on various media) than samples (i.e., 9 *A. fumigatus* strains), the data was partitioned according to the primary component of the media. More specifically, separate multiple linear regression analyses were conducted between median mouse survival and media containing MM supplemented with milk powder (MMMilk) and glucose, MMMilk and 2DG, MMMilk and acetate; between median mouse survival and minimal medium supplemented with glucose (MMG), MMG and 20 mM AA, MMG and 40 mM AA; between median mouse survival and minimal medium supplemented with lactate (MML), MML and 2DG; between median mouse survival and minimal medium supplemented with acetate (MMA), MMA and 2DG; between median mouse survival and minimal medium supplemented with mucin (MMMuc), MMMuc and 2DG; between median mouse survival and MM supplemented with gelatin (MMGel), MMGel and 2DG or between median murine survival and MM without AMM and supplemented with glucose and milk (AMMGMilk), AMMGMilk and ammonium, AMMGMilk and nitrate or AMMGMilk and urea using the “lm()” function. To determine the relative contribution of significantly correlated growth phenotypes to predicting mouse survival, the R package relaimpo, version 2.2-3 (Grömping, 2006) was applied, using the “lmg” metric of contribution.

## Results

### Phenotypic Characterization of *A. fumigatus* Clinical Isolates

**FIGURE 1:**
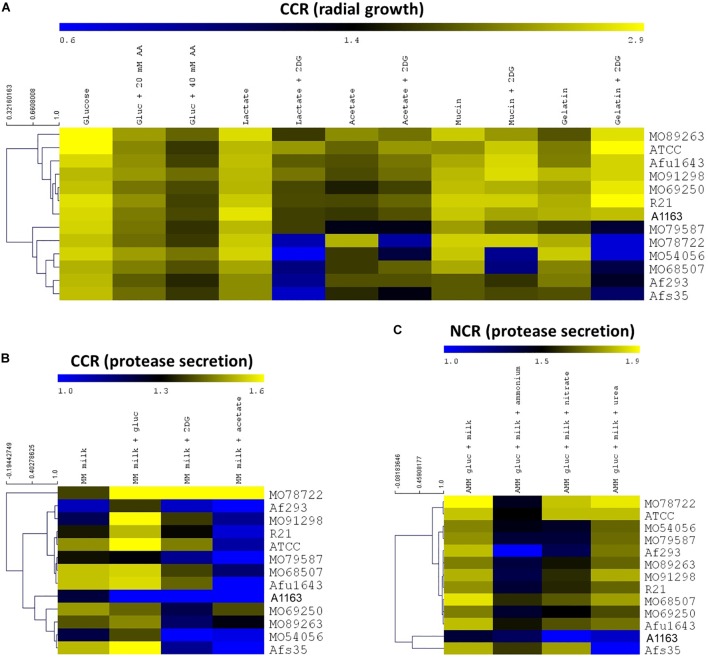
*Aspergillus fumigatus* clinical isolates show heterogeneous growth and protease secretion in carbon catabolite repressing (CCR) and nitrogen catabolite repressing (NCR) conditions. **(A)** Colony diameters of strains when grown in the presence of CCR conditions are represented as a heat map. **(B,C)** Heat maps of protease secretion halo indices when strains were grown in CCR **(B)** or NCR **(C)** conditions. Heat maps show the average of two biological replicates (MM, minimal medium; AA, allyl alcohol; 2DG, 2-deoxyglucose; AMM, minimal medium without a nitrogen source). Branches depict the closeness of strains based on the aforementioned growth or protease secretion phenotypes.

To determine whether a potential correlation exists between carbon or nitrogen catabolite repression (CCR and NCR) and virulence in *A. fumigatus*, a phenotypic characterization of 13 clinically isolated strains (Table 1), as well as of the reference strain A1163/CEA10 (chosen as reference strain as it is routinely used in laboratory conditions for basic manipulations) was first carried out. Strains were grown on MM (using nitrate as the nitrogen source) in the presence of carbon sources (glucose, acetate, lactate, mucin, and gelatin) that can potentially be used during by the fungus during host invasion (Ries et al., 2018a), and also in the presence and absence of 2-deoxyglucose (2DG) and allyl alcohol (AA) (Supplementary Table S1). Both 2DG and AA are reporters of defects in carbon catabolite repression (CCR), with 2DG not being metabolized after the second step in glycolysis, whereas AA is converted to acrolein by alcohol dehydrogenase (ADH), whose transcriptional expression is under the control of CCR (Felenbok et al., 2001). Increased resistance to 2DG and increased sensitivity to AA signifies increased CC de-repression with the strain using the carbon source other than glucose, whereas increased sensitivity to 2DG and increased resistance to AA signifies a stronger CCR. In addition, protease secretion of the same strains was also assessed in CCR and NCR conditions (Supplementary Table S1). The secretion of proteases by *A. fumigatus* during infection is predicted to be an important allergen, linked to a number of allergic conditions (Farnell et al., 2012). Furthermore, in *Aspergillus nidulans*, protease secretion is subject to CCR and NCR regulation (Katz et al., 2008) and personal observations although this has not been investigated in *A. fumigatus* yet. Protease secretion was assayed on MM supplemented with dry-skimmed milk as either the sole carbon or nitrogen source; or in combination with a competing carbon or nitrogen source and is observed as a secretion halo on these plates (Supplementary Figure S1A). Figure 1 shows, in the form of heat maps, the strain-specific colony diameters and protease secretion indexes when grown in the presence of different CCR and NCR conditions.

The clinical isolates MO79587, MO78722, MO54056, MO68507, and Afs35 are sensitive to 2DG in combination with lactate and gelatin, whereas strains MO78722 and MO54056 grew less in the presence of acetate and 2DG; and strains MO54056 and MO68507 were restricted in growth in the presence of mucin and 2DG (Figure 1A). Similar to the observed strain-specific growth restrictions in the presence of 2DG, heterogeneity in sensitivity/resistance to AA was observed for the strains, although the effects were less pronounced than in the presence of 2DG (Figure 1A). In particular, strains MO91298 and MO54056 had increased growth in the presence of AA when compared to the other strains, whereas strains ATCC 1307, MO79587, and Af293 grew less in comparison in the same conditions (Figure 1B). Similarly, when strains were grown on MM supplemented with milk powder in the presence of glucose, 2DG and acetate to assay for the effect of CCR on protease secretion in the presence of preferred and non-preferred carbon sources, strain-specific differences were observed. Strains Af293, A1163, and MO54056 generally secreted lower amounts of proteases when compared to the other strains in all tested conditions (Figure 1B). In contrast to *A. nidulans*, the presence of glucose did not have a repressive effect on *A. fumigatus* protease secretion (Ries et al., 2016) (the exception was strain A1163). Rather, the presence of alternative carbon sources such as acetate significantly reduced protease secretion in almost all tested strains (Figure 1B). Some strains, such as MO78722 and Afs35, secreted high amounts of proteases in almost all tested conditions, again highlighting the phenotypic diversity amongst these strains (Figure 1B).

Protease secretion in the presence of preferred (ammonium) and non-preferred nitrogen sources (nitrate, urea) also highlighted specific differences between strains (Figure 1C). Whereas the presence of ammonium induced NCR in most strains, as seen by a severe reduction in protease secretion, strains MO68507, Asfu1643, and Afs35 still secreted high amounts of proteases in the presence of ammonium, suggesting that NCR is less efficient in these strains (Figure 1C). Similarly, the presence of nitrate also caused a reduction in protease secretion in most strains (Figure 1C). On the other hand, the presence of urea did not have any inhibitory effect on protease secretion in all strains except for strains A1163 and Afs35, suggesting that the latter two strains are using urea efficiently as a nitrogen source when compared to the other strains (Figure 1C). In summary, these results show high variability in CCR and NCR between *A. fumigatus* clinical isolates and suggest heterogeneity in populations of clinical isolates.

### Nitrogen Catabolite Repression-Related Protease Secretion Correlates With Murine Survival

To determine whether CCR and NCR are important for *A. fumigatus* pathogenicity, the virulence of each clinical isolate was first assessed in a neutropenic murine model of invasive PA and compared to strain A1163 (Table 2 and Supplementary Figure S2). Strain Af293 was not included as the virulence of this strain was the same as the virulence of strain A1163 in a leukopenic murine model (Kowalski et al., 2016). Of the 12 strains used in this study, the 6 isolates Asfu1643, MO54056, MO68507, MO78722, MO79587, and MO91298, were less virulent than strain A1163 (Table 2 and Supplementary Figure S2). Subsequently, the murine median survival for each strain was correlated with the reduction in colony growth in the presence of carbon sources, that can potentially be used by the fungus during host invasion (Ries et al., 2018a), in combination with 2DG and AA or correlated to the protease secretion index in different CCR and NCR repressing and de-repressing conditions, by calculating the Spearman rank correlation coefficient (SRCC, cut off value >0.8 or <-0.8). Strains Asfu1643, MO91298, MO79587, and MO78722 were excluded from this analysis as their median survival (length of time from the date of infection that half of the mice are still alive) was undefined, meaning that if survival exceeds 50% at the longest time point, then median survival cannot be computed and prism reports this as undefined. No significant correlation (positive or negative) was seen between strain-specific virulence and CCR, NCR, or protease secretion with all SRCCs being either <0.8 or >-0.8 (Supplementary Table S2).

**Table 2:** Virulence data of clinical isolates when compared to A1163 in a neutropenic BALB/c murine model of invasive aspergillosis.

Strain	*P*-value (Mantel–Cox)	*P*-value (Gehan–Breslow–Wilcoxon)	Virulence (compared to A1163)
ASFU 1643	0.0105	0.0170	REDUCED
ATCC 130743	0.3132	0.3663	SAME
MO54056	0.0354	0.0366	REDUCED
MO68507	0.0354	0.0366	REDUCED
MO69250	0.2305	0.2927	SAME
MO78722	0.0015	0.0019	REDUCED
MO79587	0.0234	0.0298	REDUCED
MO89263	0.8370	0.6994	SAME
MO91298	0.0090	0.0117	REDUCED
R21	0.9422	0.8484	SAME
Afs35	0.3474	0.3887	SAME


*P-values from 2 different statistical tests are shown.*

The aim of this study was to detect a potential correlation between different CCR and NCR conditions (various variables) and virulence (one fixed determinant) and therefore a regression analysis was applied to our data. Regression analyses allow the examination of the relationship between more than two variables, studying the influence of several independent variables on one dependent variable. The regression analysis was therefore carried out between murine median survival and split into: (i) MM supplemented with milk, milk and glucose, milk and 2DG or milk and acetate; (ii) MM supplemented with glucose, glucose and 20 mM AA or glucose and 40 mM AA; (iii) lactate or lactate and 2DG; (iv) acetate or acetate and 2DG; (v) mucin or mucin and 2DG; (vi) gelatin or gelatin and 2DG; (viia) nitrogen-depleted, glucose minimal medium (AMM) supplemented with milk, with milk and ammonium, with milk and nitrate or with milk and urea. The multiple linear regression analysis was split into smaller groups, based on the nature of the substrate, because the number of variables (i.e., MMmilk, MMmilk and glucose, MMmilk and 2DG, etc.) was greater than the number of observations (acquired data points). When the number of variables approaches the number of observations, overfitting can occur which will cause erroneously detected significance (Harrel, 2001). Splitting the regression analysis into smaller groups allowed a higher number of observations than variables and thus greater statistical accuracy. No significant correlation was found between these variables, except for protease secretion in the presence of AMM supplemented with different nitrogen sources in group (viia) (Table 3). To determine if one or more of the nitrogen sources (milk, ammonium, urea, or nitrate) contributed to the correlation, each nitrogen source was analyzed separately and protease secretion in the presence of (viib) AMM supplemented with milk or milk and nitrate were found to have a high correlation with median murine survival. Correlation between murine median survival and protease secretion in these two conditions resulted in a *P*-value = 0.0001, with an *R*^2^ value of 0.9522 and an *F*-statistic of 59.73 (Table 3). Furthermore, the coefficient estimate and *t*-values were calculated for the significant correlation, as both values provide directionality of the association and are informative when they significantly deviate from the null expectation. The coefficient estimate and *t*-values for AMM supplemented with milk were positive (3.39156 ± 0.31443 and 10.786, respectively), suggesting that increased protease secretion (when comparing the secretion index amongst strains) correlates with an increase in virulence; whereas both values in the presence of AMM supplemented with milk and nitrate were negative (-1.76548 ± 0.17842 and -9.895, respectively), indicating that a reduction in protease secretion (when comparing the secretion index amongst strains) in this particular NCR condition correlated with increased virulence. These results suggest that the degree of NCR in protease secretion plays an important role during mammalian host infections.

**Table 3:** Regression analysis between murine median survival and: (i) minimal medium supplemented with milk, milk and glucose, milk and 2DG or milk and acetate; (ii) minimal medium supplemented with glucose, glucose and 20 mM AA or glucose and 40 mM AA; (iii) lactate or lactate and 2DG; (iv) acetate or acetate and 2DG; (v) mucin or mucin and 2DG; (vi) gelatin or gelatin and 2DG; (viia) nitrogen-depleted, glucose minimal medium (AMM) supplemented milk, milk and ammonium, milk and nitrate or milk and urea; (viib) AMM supplemented with milk and AMM supplemented with milk and nitrate.

Condition	*R*^2^-value	*F*-statistic	*P*-value	Type of CCR/NCR
(i)	0.5530	1.2370	0.420800	CCR protease secretion
(ii)	0.2951	0.6979	0.592200	CCR radial growth
(iii)	0.3333	1.5000	0.296300	CCR radial growth
(iv)	0.0081	0.0247	0.975700	CCR radial growth
(v)	0.3630	1.7100	0.258400	CCR radial growth
(vi)	0.2580	1.0430	0.408500	CCR radial growth
(viia)	0.9647	27.3300	0.003651	NCR protease secretion
(viib)	0.9522	59.7300	0.000109	NCR protease secretion


### Metabolic Comparison Between A1163 and Afs35

**FIGURE 2:**
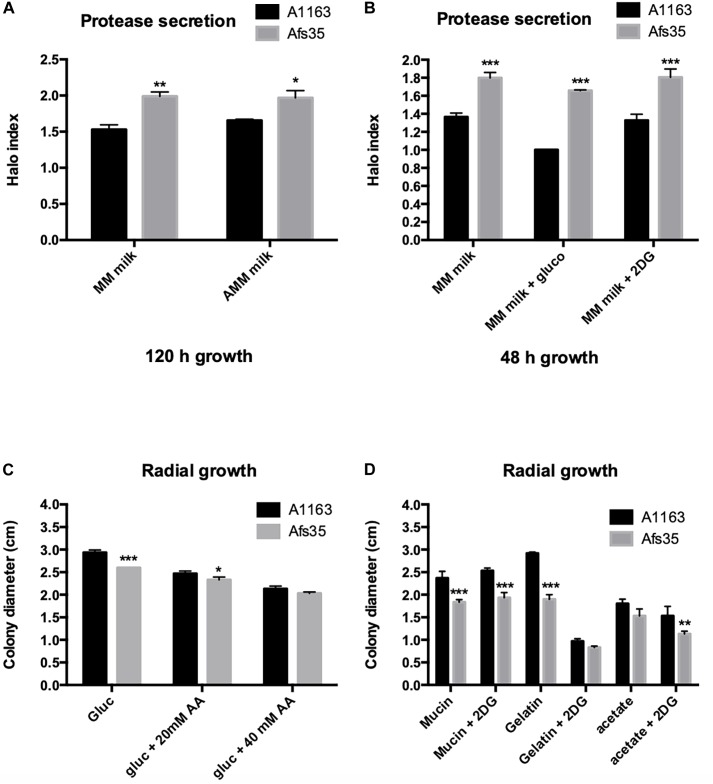
Afs35 presents increased protease secretion when compared to strain A1163. **(A,B)** Protease secretion index (normalized by colony diameter) when strains were grown for 5 days on minimal medium (MM) or MM without any nitrogen source (AMM) supplemented with dry-skimmed milk and glucose or 2-deoxyglucose (2DG). **(C,D)** Colony diameter of strains Afs35 and A1163 when grown for 48 h on MM supplemented with glucose and increasing concentrations of allyl alcohol (AA) or in the presence of different carbon sources supplemented with 2-deoxyglucose (2DG). Error bars indicate standard deviations for three biological triplicates (^∗^*P*-value < 0.05, ^∗∗^*P*-value < 0.005, ^∗∗∗^*P*-value < 0.0005 as determined by a two-way ANOVA test).

The aforementioned phenotypic analysis demonstrated that strain Afs35 secreted higher amounts of proteases in different NCR conditions when compared to strain A1163 (Figure 1) but no difference in virulence was observed. Proteases are predicted to play an important role in inflammation and lung epithelial cell disruption (Kogan et al., 2004; Namvar et al., 2015) and to further investigate the observed phenotype, protease secretion was measured in both strains when grown for 48 h in MM supplemented with milk and glucose or 2DG; or when grown for 5 days on MM supplemented with milk in the presence or absence of nitrate (using milk as either the carbon or as the carbon and nitrogen source). In agreement with the aforementioned phenotypic data, Afs35 secreted significantly more proteases in both conditions than A1163 (Figure 2A,B). Furthermore, significant growth differences were observed between both strains (Figure 2C,D). In general, strain Afs35 grew less than A1163 on all carbon sources, and in agreement with Figure 1, the presence of AA or 2DG significantly reduced growth, with the exception of mucin and 2DG, of both strains (Figure 2C,D). Nevertheless, the growth inhibitory effect exerted by AA (growth on glucose) and 2DG (growth on gelatine and acetate) were similar in both strains, indicating that CCR was similar in both strains.

**FIGURE 3:**
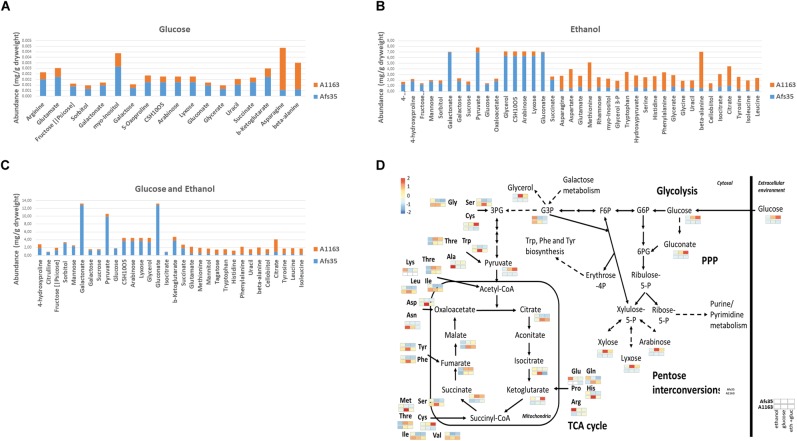
Strain Afs35 is metabolically different from strain A1163. **(A–C)** Abundance (the value of each replicate was divided by the median of the given metabolite) of quantitatively significant different metabolites from five biological replicates identified in both strains when grown for 16 h in minimal medium (MM) supplemented with glucose, ethanol or glucose and ethanol. **(D)** Diagram of metabolites of the central carbon metabolic pathways glycolysis, citric acid cycle and pentose phosphate pathway. Shown is the relative abundance of metabolites from five biological replicates, in the form of a heat map, identified in strains Afs35 and A1163 in each of the aforementioned conditions.

**FIGURE 4:**
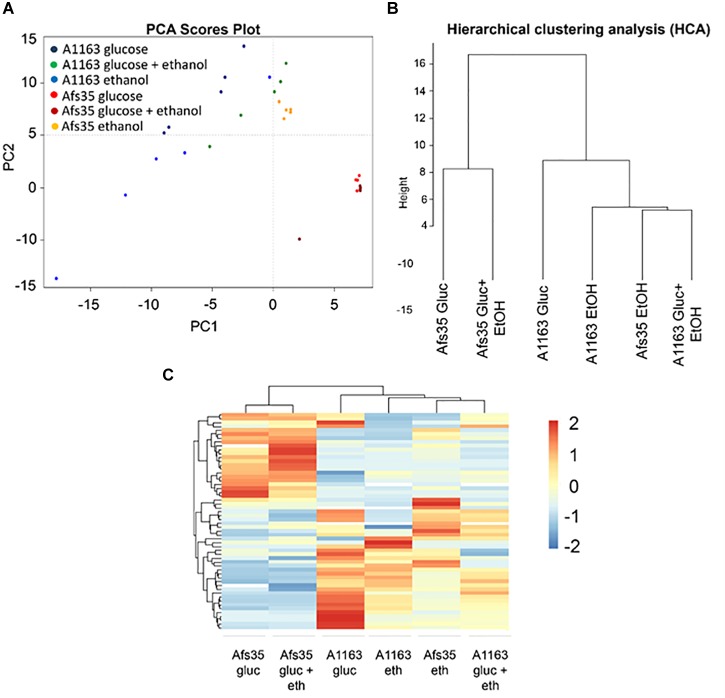
**(A)** Principal component analysis (PCA) of all five replicates and **(B)** hierarchical clustering analysis (HCA) based on the average of five replicates of the quantities of all significantly different metabolites identified between strains Afs35 and A1163 in each of the three above conditions. **(B)** HCA shows the proximity relationship between strains and conditions based on the average quantity of identified metabolites. **(C)** Heat map based on the relative average quantities of five biological replicates of all significantly different metabolites identified between strains Afs35 and A1163 when cultivated in glucose, ethanol or glucose and ethanol.

To further investigate these strain-specific growth differences, metabolome analysis was carried out when both strains were grown in the presence of glucose, ethanol and glucose and ethanol (Supplementary Table S3). Glucose is the preferred carbon source for *Aspergillus* spp. and was shown to be present in low concentrations in mammalian lung tissue (Beattie et al., 2017), whereas ethanol is an alternative carbon source and is produced by *A. fumigatus* during adaptation to lung hypoxic environments (Grahl et al., 2011). In all three conditions, intracellular levels of different amino acids were lower in strain Afs35, whereas the here identified TCA cycle intermediates and intracellular sugars, such as glucose, galactose, mannose and arabinose, were significantly increased in this strain when compared to strain A1163 (Figure 3A–D). In agreement, PCA (principal component analysis) and HCA (hierarchical clustering analysis) plots showed that A1163 and Afs35 are metabolically different from each other in all three carbon sources, except for ethanol, where both strains had a related metabolic profile (Figure 4A,B). The metabolic profiles of Afs35 cultivated in glucose or glucose and ethanol are closely related and different from Afs35 grown in ethanol only (Figure 4C). The latter profile is more similar to the profiles of A1163 cultivated in ethanol or glucose and ethanol (Figure 4C). The aforementioned results highlight fundamental differences in primary carbon metabolism between both strains which were not due to a de-regulated ADH (alcohol dehydrogenase) activity, despite strain-specific variations, with strain Afs35 having higher ADH activity in the presence of ethanol or ethanol and glucose (Supplementary Figure S1B). The aforementioned differences in metabolism between strains Afs35 and A1163 may be the result of short nucleotide polymorphisms (SNPs), that incorporate base pair substitutions, InDels (insertion–deletions), where insertion or deletion of one or more base pairs occurs, and/or CNVs (copy number variations), in which genes or sections of the genome are repeated, as has previously been described for different *A. fumigatus* isolates (Lind et al., 2017).

### Genomic Comparison of Afs35 and A1163

Subsequently, the genome of Afs35 was sequenced and compared to the genome of A1163. Mapping of the Afs35 reads onto the A1163 genome assembly identified a total of 13,592 SNPs between the two strains, with 3,871 SNPs occurring within predicted gene coding sequences (Supplementary Table S4). Furthermore, 313 small-scale indels (insertion–deletions) were detected in the genome of Afs35 of which 119 indels occurred within predicted gene coding sequences (Supplementary Table S4). In addition, 40 large-scale CNVs (copy number variations, pre-dominantly deletions) were observed that span a region of 1,215,960 bp, with 266 gene deletions occurring in strain Afs35 (Supplementary Table S4). Differences between both genomes are summarized in Table 4. Based on these genome mutational data, the genomes of Afs35 and A1163 diverge by 4.212%.

**Table 4:** Identified mutations in the genome of Afs35 when compared to the genome of A1163 (SNP, single nucleotide polymorphism; CNV, copy number variation).

Mutation type	Total	Coding region-specific
SNP	13592	3871
Insertions	149	59
Deletions	164	60
CNV	40	267


*Shown are the total number of mutations and the number of mutations predicted to be within gene coding regions.*

Furthermore, the severity of mutations on gene function was predicted and categorized as high, moderate and low impact mutations (Supplementary Table S5). Low impact mutations included synonymous base pair substitutions that had no effect on the type of encoded amino acid and in base pair changes that occurred within introns or the 5′ UTR (untranslated) region of the gene. Moderate mutations included all missense mutations, where a base pair substitution resulted in an amino acid change in the sequence of the corresponding protein. High impact mutations encompassed all gene mutations that resulted in a premature stop codon, in the loss of the gene start codon or in changes in the genes’ reading frame (frameshift). In total, 78 high impact mutations and 2,827 moderate impact mutations were found in the genome of Afs35 when compared to A1163 (Supplementary Table S5).

Based on our earlier phenotypic and metabolic data, high and moderate impact mutations (low impact mutations were discarded for further analysis) were screened for genes encoding enzymes of carbon and amino acid metabolism as well as genes encoding proteins involved in protease secretion and cell wall metabolism (Supplementary Table S6).

Several genes encoding enzymes that are important for central carbon metabolic pathways such as glycolysis (e.g., the pyruvate dehydrogenase kinase PkpA, AFUB_029240), gluconeogenesis, the citric acid cycle (CAC) (e.g., a putative isocitrate dehydrogenase, AFUB_034080) and pyruvate utilization (e.g., the pyruvate decarboxylase PdcC, AFUB_062480) have accumulated at least one missense mutation in Afs35 (Supplementary Table S6). Furthermore, missense mutations were found in the alcohol dehydrogenase-encoding gene *alcC*, the transcription factor-encoding gene *facB* and the acetyl-CoA synthetase-encoding gene *facA* (Supplementary Table S6). AlcC has previously been shown to be required for ethanol utilization and suggested to play a role in the pathogenesis of invasive PA (Grahl et al., 2011). In contrast, FacA and FacB are important for acetate utilization, based on comparison with their homologs in *A. nidulans* (Todd et al., 1998), although they remain uncharacterized in *A. fumigatus*.

In addition, 2 genes encoding enzymes required for galactose and cell wall sugar precursor metabolism also had at least one missense mutation in strain Afs35. These included the UDP-glucose-4-epimerase Uge5 and an additional putative UDP-galactose-4-epimerase (Afu7g00360). Uge5 has previously been shown to be required for the synthesis of galactofuranose, a component of fungal cell wall galactomannan and to contribute galactose for the synthesis of galactosaminogalactan (Lee et al., 2014), whereas Afu7g00360 has not been characterized to date. Furthermore, several genes encoding enzymes involved in cell wall glucan and chitin metabolism, such as the α-1,3-glucan synthases Ags1, 2, and 3, the exo- and endo-β-1,3-glucanases Exg2, 10, 19, and Eng5 as well as the chitin synthases ChsA, CsmB, ChiA1, and Chi1 had at least one or two missense mutations in the open reading frame (ORF) (Supplementary Table S6).

Finally, strain Afs35 also had one or more missense mutations in genes encoding enzymes required for the uptake and metabolism of various nitrogen sources (Supplementary Table S6). These included enzymes of metabolic pathways required for the utilization and/or synthesis of different amino acids, the transport and utilization of nitrate and the metabolism of urea. Furthermore, high impact mutations were also observed for 2 genes, including the loss of a stop codon in gene AFUB_034430, encoding the homolog of *Saccharomyces cerevisiae* Aro1p and a frameshift mutation in the urease-encoding gene AFUB_004900 (Supplementary Table S6). Aro1p is required for the synthesis of chorismate, a precursor for all aromatic amino acids (Duncan et al., 1987). In addition, the transcriptional regulator of protease secretion PrtT (Sharon et al., 2009) has a missense mutation resulting in the substitution of isoleucine at position 441 by leucine (Supplementary Table S6).

In summary, the genome of Afs35 contains several different mutations in genes required for the utilization of different carbon and nitrogen sources as well as for the biosynthesis of cell wall polysaccharides when compared to strain A1163.

### Afs35 and A1163 Differ in Their Cell Wall Organization and Composition in the Presence of Glucose

**FIGURE 5:**
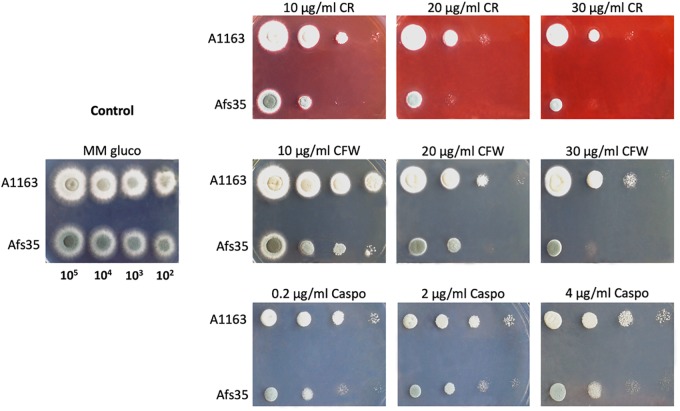
*Aspergillus fumigatus* strain Afs35 is more sensitive to cell wall disturbing compounds than strain A1163. Tenfold serial dilutions of strains when grown for 48 h on MM (minimal medium) supplemented with glucose and increasing concentrations of congo red (CR), calcofluor white (CFW) and caspofungin (CF).

The high number of mutational differences between Afs35 and A1163 in genes encoding cell wall remodeling enzymes as well as an accumulation of intracellular sugars, as determined by metabolome analysis, that can serve as precursors for cell wall polysaccharides (Engel et al., 2012) in Afs35, may hint at a difference in cell wall composition between the two strains. To test this hypothesis, both strains were grown on glucose-containing MM supplemented with different concentrations of the cell wall perturbing agents caspofungin (β-glucan synthase inhibitor), congo red (glucan-binding molecule) and CFW (chitin-binding molecule). Strain Afs35 was more sensitive to these agents than A1163, suggesting differences in cell wall composition and/or organization when compared to A1163 (Figure 5).

To further investigate potential differences in cell wall composition and/or organization, cell wall-associated proteins from conidia of both strains were extracted, identified and quantified by mass spectrometry. *A. fumigatus* conidia are primary infectious agents that when inhaled, adhere to respiratory tract epithelial cells where they can initiate host colonization, dependent on the immune status of the subject (Croft et al., 2016). A total of 75 proteins were identified (Supplementary Table S7), but only four cell wall-associated proteins had a significant statistical difference (*p*-value < 0.05) in quantity between both Afs35 and A1163 strains. The four proteins included two (AFUB_024920, DppV) dipeptidyl-peptidases (DPP, AFUB_024920 had ∼3-fold reduction in Afs35, whereas DppV was not detected in Afs35), a β-glucosidase (∼7.5-fold increase in Afs35) and an α-1,2-mannosidase (∼2-fold increase in Afs35) (Supplementary Table S7). The genome of *A. fumigatus* is predicted to encode two secreted DPPs (Maeda et al., 2016), DppV and DppVI, with only DppV being identified here on the conidial surface. DppV has previously been shown to be an immune-reactive protein and was identified in patients with invasive aspergillosis (Shi et al., 2012). The β-glucosidase Exg13 (encoded by Afu7g06140) and α-1,2-mannosidase MsdS (encoded by Afu1g14560) identified in our mass spectrometry studies are predicted to be involved in cell wall remodeling (Mouyna et al., 2013). Moreover, the α-1,2-mannosidase MsdS is immunogenic and predicted to bind to the human protein fibrinogen (Kumar et al., 2011; Upadhyay et al., 2012). These results suggest quantitative differences in the conidia cell wall-associated proteins that are predicted to be immunoreactive.

### Virulence of A1163 and Afs35 Differs Based on the Type of Mouse Model

**FIGURE 6:**
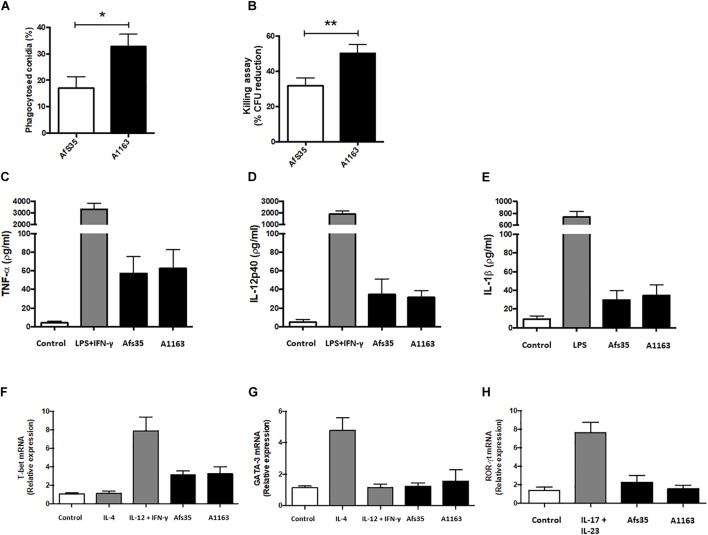
Strain Afs35 is more resistant to *in vitro* macrophage-mediated killing than strain A1163 but does not elicit a different immunological response. Bone marrow-derived macrophages (BMDMs) were obtained from C57BL/6 adult mice. Phagocytosis index **(A)** and killing activity **(B)** is increased in strain A1163 when statistically compared to strain Afs35. To evaluate the level of relevant cytokines, BMDMs were incubated with culture medium, LPS (lipopolysaccharides) + INFγ, and *A. fumigatus* strains Afs35 or A1163. The supernatants were assayed for TNF-α **(C)**, IL-12p40 **(D)**, and IL-1β **(E)** levels and comparisons were made between the control condition and both strains. The levels of relative mRNA expression of T-bet **(F)**, GATA-3 **(G)**, and ROR-γt **(H)** were determined by real-time PCR, using the β-actin gene as a control comparing the control condition with both strains. The results represent the mean ± SD (standard deviation) from three independent biological replicates. ^∗^*p* < 0.05, ^∗∗^*p* < 0.005 from an unpaired, equal variance student’s *t*-test, with strain Afs35 compared to strain A1163 **(A,B)**, or from a one-way ANOVA test with multiple comparisons **(C–H)**.

The fungal cell wall has been shown to be crucial for interacting with the mammalian immune system and has been attributed immune-evasive and –modulatory properties (Chai et al., 2011; Cramer et al., 2011). Subsequently, the immunological response elicited by both strains was tested *in vitro* in the presence of murine BMDM. Conidia of strain Afs35 were less phagocytised and this strain had increased survival after exposure to macrophages when compared to strain A1163 (Figure 6A,B). Both strains elicited an immunological response as observed by increased levels of TNF (tumor necrosis factor)-α (Figure 6C), IL-12p40 (Figure 6D), IL (interleukin)-1β (Figure 6E), and expression of the transcription factor T-bet (Figure 6F) when compared to the control conditions, although no differences between both strains in this response and in the expression of GATA-3 (Figure 6G) and ROR-γt (Figure 6H) were observed. These results suggest that strain Afs35 is less susceptible to macrophage-mediated killing *in vitro*.

**FIGURE 7:**
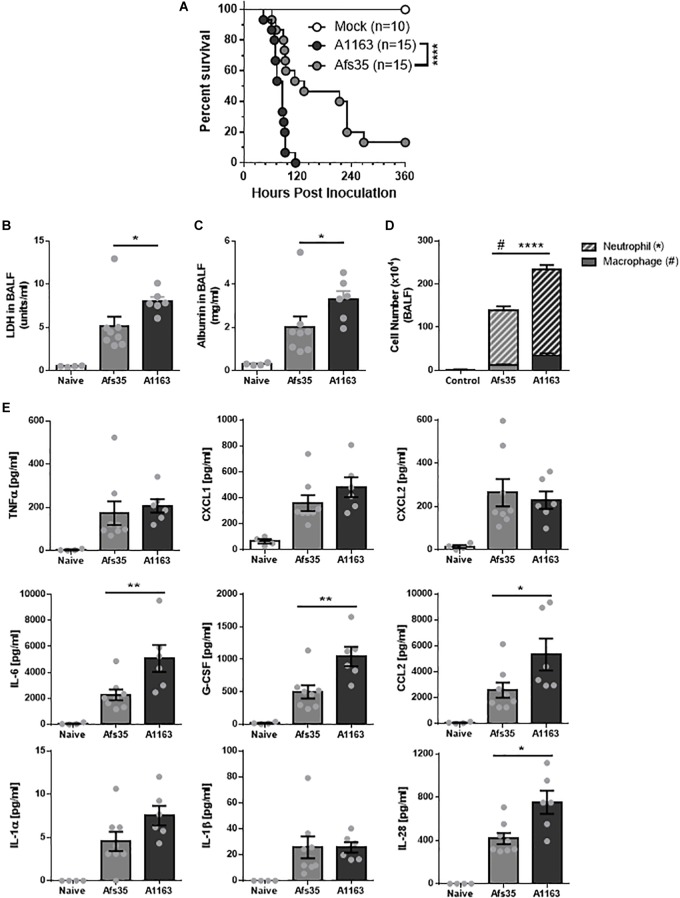
Strain A1163 is more virulent than strain Afs35 in a triamcinolone murine model of pulmonary aspergillosis (PA). **(A)** Survival curve of triamcinolone-treated mice infected with phosphate buffered saline (PBS = mock, control) and strains A1163 and Afs35 for 15 days. 15 (*n*) mice were infected with each strain and log-rank test was used (^∗∗∗∗^*p* < 0.0001) to assess statistical differences. **(B)** Lactate dehydrogenase activity, **(C)** albumin concentrations, and **(D)** number of macrophages and neutrophils in bronchoalveolar lavage fluid (BALF) 72 h after mice were infected with PBS (naïve) or strains A1163 and Afs35 (^#^*p* < 0.05 refers to macrophage numbers and ^∗∗∗∗^*p* < 0.0001 refers to neutrophil numbers). **(E)** Chemokine and cytokine profile of BALF and lung homogenate in mice 72 h post-infection with either PBS (naïve), strain A1163 or strain Afs35. Error bars indicate the mean ± SD (standard deviation) from three independent biological replicates. ^∗^*p* < 0.05, ^∗∗^*p* < 0.01, ^∗∗∗^*p* < 0.001, and ^∗∗∗∗^*p* < 0.0001 from a one-way ANOVA test with Dunn’s post-test.

These results suggest that there may be strain-specific differences in pathogenesis, although virulence of both strains in a neutropenic mouse model was the same (Table 2 and Supplementary Figure S2). To determine whether differences in virulence between A1163 and Afs35 exist in a non-neutropenic mouse model, triamcinolone-treated mice were subjected to infection with A1163 and Afs35. Strain A1163 killed all mice after 5 days (120 h) whereas Afs35 was significantly attenuated in virulence and ∼15% of mice survived after 15 days post-infection (Figure 7A). Lactate dehydrogenase (LDH) activity (Figure 7B) and albumin concentrations (Figure 7C), which are indicators of lung damage and leakage, respectively, were significantly greater in BALF from A1163 challenged mice compared with Afs35 challenged mice, suggesting that strain A1163 induced greater lung damage. Additionally, mice challenged with strain A1163 had significantly higher numbers of neutrophils and macrophages in the BALF than Afs35 challenged mice (Figure 7D). The levels of the pro-inflammatory interleukins IL-6 and IL-28, the chemokine ligand-2 (CCL2) and the granulocyte colony-stimulating factor (G-CSF) were also significantly higher in mice challenged with A1163 than with strain Afs35, indicating that strain A1163 stimulated a greater inflammatory response (Figure 7E). In agreement with the aforementioned *in vitro* data, TNF-α and IL-1β concentrations were not significantly different in mice infected with either strain (Figure 7E). Together, these results indicate that strain A1163 is more virulent than strain Afs35 in a triamcinolone murine model of invasive aspergillosis.

## Discussion

*Aspergillus fumigatus* is an opportunistic human fungal pathogen and its virulence is determined by a range of factors (Supplementary Table S8), such as germination rate, spore pigmentation, growth in hypoxic environments, cell wall stability and maintenance as well as the ability to use and metabolize a variety of carbon and nitrogen sources during mammalian host infection. The role of carbon and nitrogen metabolism in *A. fumigatus* pathogenicity is based largely on studies that have focused on characterizing the virulence of strains deleted for one metabolic enzyme-encoding gene (Krappmann and Braus, 2005; Rhodes, 2006; Willger et al., 2009; Ries et al., 2018a), whereas the relationship between the ability to use different carbon or nitrogen sources and virulence has not been investigated. This work therefore aimed at investigating a potential correlation between the virulence of 13 *A. fumigatus* clinical isolates in a neutropenic mouse model and carbon or nitrogen catabolite repression (CCR and NCR). CCR and NCR are metabolic processes that allow the utilization of preferred carbon (e.g., glucose) and nitrogen (e.g., ammonium and glutamine) sources, therefore providing quick energy and material for niche colonization and survival (Ruijter and Visser, 1997; Koon et al., 2008).

Great heterogeneity exists in the degree of CCR and NCR in all 13 clinical isolates. This agrees with previous observations that identified a great level of genetic diversity within *A. fumigatus* isolates (Duarte-Escalante et al., 2009; Launay and Dunbar, 2015). Our study did not find a correlation between CCR and virulence, although the existence of such a correlation cannot be excluded considering the sample size used in this study. Nevertheless, our sample size should be big enough to detect a potential correlation between the aforementioned factors, as a previous study found a correlation between *A. fumigatus* virulence and fitness in hypoxia, using 14 environmental and clinical isolates (Kowalski et al., 2016). Furthermore, this work found a positive correlation between the effect of NCR on protease secretion and virulence. Protease secretion on nitrogen-depleted MM where milk powder or milk powder and nitrate were the sole nitrogen sources, correlated positively with virulence. In addition to the preferred nitrogen source ammonium, the presence of nitrate also resulted in NCR and a reduction of protease secretion, possibly indicating the preferential utilization of nitrate by *A. fumigatus* whose primary nitrogen source in the soil is nitrate (Krappmann and Braus, 2005). In *Candida albicans*, the amount and type of nitrogen sources have been shown to influence virulence determinants such as cellular morphogenesis and the ability to modify the pH of the extracellular environment, therefore ensuring successful infection and survival within the host environment (Biswas and Morschhäuser, 2005; Vylkova and Lorenz, 2014). *A. fumigatus* is predicted to face nitrogen starvation during infection establishment as was shown by an up-regulation of genes required for amino acid transport and biosynthesis (McDonagh et al., 2008). Accordingly, the biosynthesis of lysine and the cross-pathway control system, that controls the response to amino acid starvation, have been shown to be crucial for *A. fumigatus* virulence (Krappmann and Braus, 2005). These aforementioned studies highlight the importance of nitrogen acquisition and metabolism during infection and support the here reported findings on the significance of nitrogen utilization during mammalian host invasion. In agreement with (Ries et al., 2018a), NCR, or at least components of this metabolic process, may be important for pathogenicity, although additional studies are required to elucidate the exact role of NCR in *A. fumigatus* virulence.

Proteases are crucial for nitrogen acquisition and are predicted to be important for breaching the alveolar epithelial cell barrier and promoting host tissue inflammation (Kogan et al., 2004; Mavor et al., 2005; Namvar et al., 2015). Among the 13 *A. fumigatus* clinical isolates used in this study, strain Afs35 was selected for further characterization as it secreted high amounts of proteases under NCR conditions. Metabolite quantification of Afs35 and comparison to the reference strain A1163 during growth in physiologically relevant carbon sources showed substantial differences in intracellular amino acid and sugar levels between both strains. Strain Afs35 consistently presented lower quantities of several different amino acids and an accumulation of simple sugars when compared to strain A1163 in all growth conditions. In addition, both the alcohol dehydrogenase activity, which was higher in strain Afs35 in the presence of ethanol or ethanol and glucose when compared to strain A1163, and the high secretion of proteases by strain Afs35 in the presence of NCR and CCR conditions, indicate essential differences in primary metabolism between both strains, although the exact mechanistic nature underlying these processes remains to be confirmed. Furthermore, Afs35 genome sequencing and subsequent mutation analysis detected a missense mutation in the transcription factor PrtT, the master regulator of genes encoding proteases (Bergmann et al., 2009; Sharon et al., 2009; Hagag et al., 2012; Shemesh et al., 2017). Whether this missense mutation is responsible for the observed increase in protease secretion remains to be determined. In addition, missense and high impact mutations were found in genes encoding enzymes required for carbohydrate and amino acid biosynthesis or utilization. Afs35 genome analysis is in agreement with the observed phenotypic and metabolome findings, although additional studies on these mutations are required for confirmation. Alternatively, mutations that are not located within genes encoding enzymes of carbon and nitrogen metabolism may also contribute to the observed phenotypes and influence the degree of NCR in strain Afs35.

Intracellular simple sugars can serve as precursors for cell wall polysaccharides (Engel et al., 2012), and strain Afs35 had higher quantities of these sugars when compared to strain A1163. Indeed, strain Afs35 was more sensitive to cell wall perturbing agents when compared to strain A1163, suggesting differences in cell wall composition and/or organization between both strains. In addition, the Afs35 genome analysis found a substantial amount of missense mutations in genes encoding enzymes involved in cell wall chitin and glucan biosynthesis, which could, at least partially, be responsible for the observed phenotypes. The severity and impact on cell wall structure and/or organization in Afs35 remain to be determined but analysis of conidial surface proteins between both strains found 2 cell wall remodeling enzymes being present in higher quantities in Afs35 conidia, further supporting differences in cell wall structure and/or composition between both strains.

The observed, significant differences in genome sequences between strains Afs35 and A1163 further support the existence of great heterogeneity that exists between *A. fumigatus* strains and is in agreement with previous studies that analyzed the relationship of genome sequences from different isolates (Lind et al., 2017). It is noteworthy that deletion of Afs35 *akuA* (KU70) could result in some of the observed genome mutations due to defects in the non-homologous end-joining pathway and subsequent increased genome instability although to date no major phenotypic differences with the respective parental strain ATCC 46645 have been described. In addition, the number of SNPs detected between Afs35 and A1163 (∼13,500) was roughly 2.5-fold less than the average number of SNPs (∼50,000) previously described for different *A. fumigatus* clinical isolates (Knox et al., 2016; Rosowski et al., 2018). This suggests less genetic diversity between strains Afs35 and A1163 when compared to differences in genome between other *A. fumigatus* clinical isolates (Knox et al., 2016; Rosowski et al., 2018). Next, the impact of these metabolome and genomic results on virulence were further investigated. No difference in virulence between strains Afs35 and A1163 was detected in a neutropenic mouse model, which led to the question whether strain virulence could change depending on the mammalian host immune status, especially as the genomic data suggests substantial differences in important virulence factors, such as nitrogen metabolism and cell wall structure/composition, between both strains. *In vitro* phagocytosis and killing assays of conidia from strains Afs35 and A1163 by BMDMs showed that strain Afs35 was less phagocytised and had a higher survival rate after BMDM passage. Additional *in vivo* experiments, where triamcinolone-treated mice were challenged with both *A. fumigatus* strains, showed that strain Afs35 was attenuated in virulence when compared to strain A1163. These results were further corroborated by an increase in pulmonary damage than when compared to strain Afs35. The discrepancies between these results and the results obtained with neutropenic mice are likely due to the type of immune suppression used as well as substantial differences between *in vivo* and *in vitro* assays. In addition, *in vivo* treatments are likely to have a different influence on macrophage and neutrophil activity than when compared to the *in vitro* assays, which ultimately could also influence the outcome of strain-specific virulence. In agreement, other studies have found differences in virulence between strains that were dependent on the used mouse model (Fuller et al., 2016; Kowalski et al., 2016; Supplementary Table S8). In addition, it is known that the severity of *A. fumigatus* infections depends on the status of the mammalian immune system (Abad et al., 2010). It is therefore crucial to use various mouse/animal models when assessing virulence of different strains as strain pathogenicity is likely to change depending on the fitness of the host’s immune system.

## Conclusion

This study further emphasizes the importance of nitrogen acquisition and metabolism during *A. fumigatus* infection and highlights the great genetic heterogeneity that exists between strains. In addition, attention is drawn to the importance of using different mouse/animal models to correctly assess differences in strain-specific virulence in future studies.

## Ethics Statement

The principles that guide our studies are based on the Declaration of Animal Rights ratified by the UNESCO on January 27, 1978, in its eighth and 14th articles. All protocols used in this study were approved by the local ethics committee for animal experiments from the Campus of Ribeirão Preto, Universidade de São Paulo (Permit Number: 08.1.1277.53.6; Studies on the interaction of *A. fumigatus* with animals) or the Dartmouth College Institutional Animal Care and Use Committee (Protocol Number: 00002168). All animals were housed in groups of five within individually ventilated cages and were cared for in strict accordance with the principles outlined by the Brazilian College of Animal Experimentation (Princípios Éticos na Experimentação Animal—Colégio Brasileiro de Experimentacão Animal, COBEA) and Guiding Principles for Research Involving Animals and Human Beings, American Physiological Society. All efforts were made to minimize suffering. Animals were clinically monitored at least twice daily and humanely sacrificed if moribund (defined by lethargy, dyspnoea, hypothermia, and weight loss). All stressed animals were sacrificed by cervical dislocation.

## Author Contributions

LR, GG, JO, and AR designed the experiments. LR, JS, PC, PL, FA, LA, AM, AT-N, YK, DH, HT, XW, and JO performed the experiments. LR, JS, JO, and AR analyzed the data. LR, LA, and GG wrote the manuscript.

## Conflict of Interest Statement

The authors declare that the research was conducted in the absence of any commercial or financial relationships that could be construed as a potential conflict of interest.

## Footnotes

1 http://boevalab.com/FREEC/

2 https://en.wikipedia.org/wiki/Spearman%27s_rank_correlation_coefficient
