# Supplementary material for: Nutritional Heterogeneity Among Aspergillus fumigatus Strains Has Consequences for Virulence in a Strain- and Host-Dependent Manner
Source: Front Microbiol. 2019 Apr 24;10:854. doi: 10.3389/fmicb.2019.00854 (PMC6492530; doi:10.3389/fmicb.2019.00854)
Supplement: Supplementary file 9 [file Data_Sheet_1.PDF]

A. **B.**

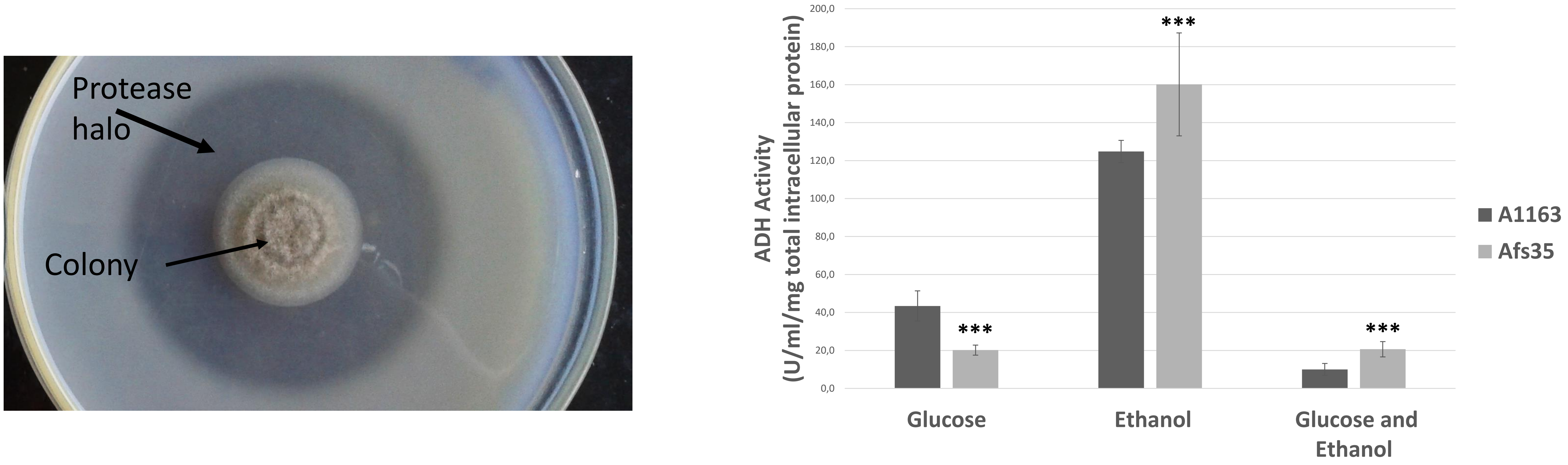

Figure S1. A. Protease secretion of strain Afs35 when grown for 5 days at 37°C on minimal medium supplemented with dry-skimmed milk. Protease halo and colony are depicted by arrows. **B**. Alcohol dehydrogenase activity (ADH) when strains were grown for 16 h in minimal medium supplemented with glucose, ethanol or glucose and ethanol. Enzyme activity was normalised by intracellular protein concentration.

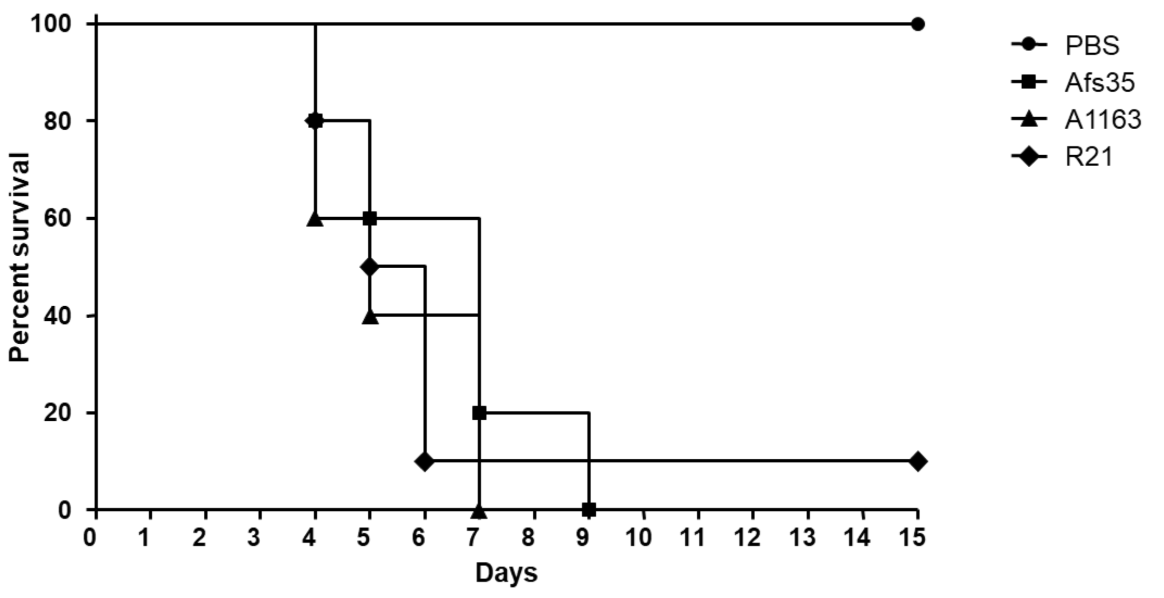

Median survival
Afs35 7.000
A1163 5.000

Log-rank (Mantel-Cox) Test
P value 0.3474
P value summary ns
Are the survival curves sig different? No

Gehan-Breslow-Wilcoxon Test
P value 0.3837
P value summary ns
Are the survival curves sig different? No

Median survival
A1163 5.000
R21 5.500

Log-rank (Mantel-Cox) Test
P value 0.9422
P value summary Ns
Are the survival curves sig different? No

Gehan-Breslow-Wilcoxon Test
P value 0.8484
P value summary ns
Are the survival curves sig different? No

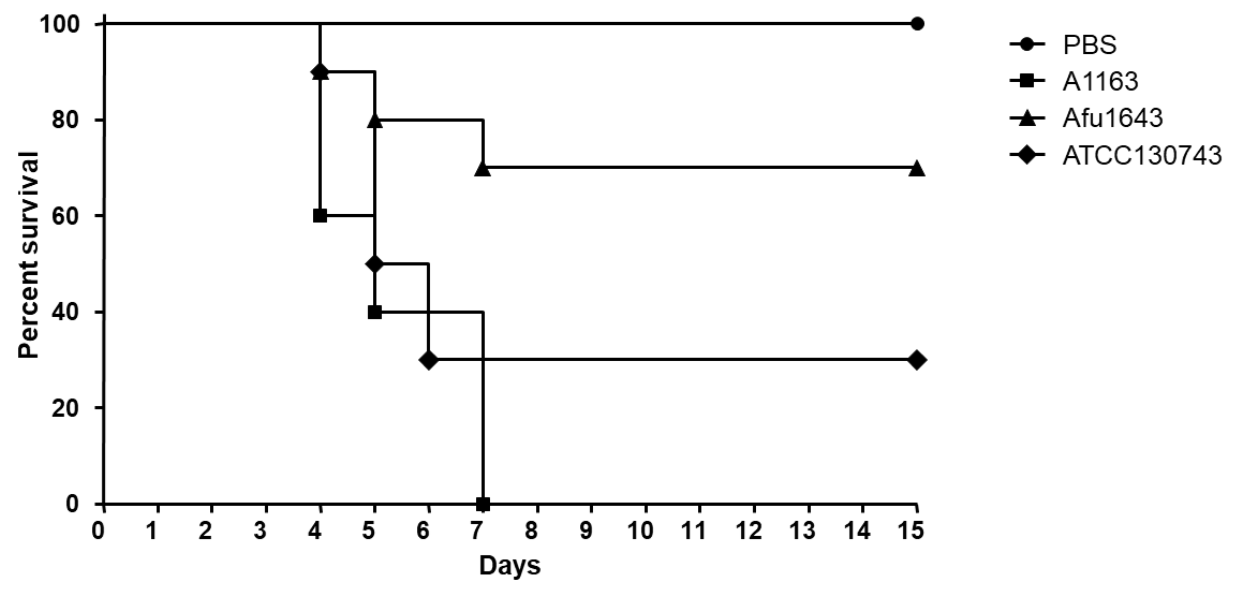

Median survival
A1163 5.000
Afu1643 Undefined

Log-rank (Mantel-Cox) Test
P value 0.0105
P value summary *
Are the survival curves sig different? Yes

Gehan-Breslow-Wilcoxon Test
P value 0.0170
P value summary *
Are the survival curves sig different? Yes

Median survival
A1163 5.000
ATCC130743 5.500

Log-rank (Mantel-Cox) Test
P value 0.3132
P value summary ns
Are the survival curves sig different? No

Gehan-Breslow-Wilcoxon Test
P value 0.3663
P value summary ns
Are the survival curves sig different? No

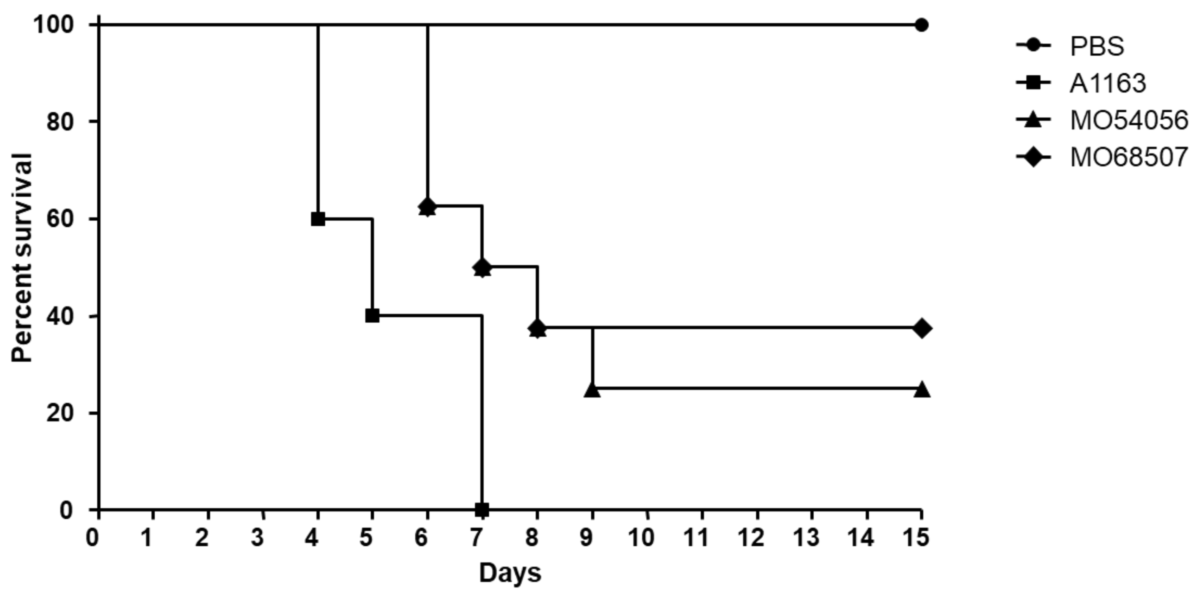

Median survival
A1163 5.000
MO54056 7.500

Log-rank (Mantel-Cox) Test
P value 0.0354
P value summary *
Are the survival curves sig different? Yes

Gehan-Breslow-Wilcoxon Test
P value 0.0366
P value summary *
Are the survival curves sig different? Yes

Median survival
A1163 5.000
MO68507 7.500

Log-rank (Mantel-Cox) Test
P value 0.0354
P value summary *
Are the survival curves sig different? Yes

Gehan-Breslow-Wilcoxon Test
P value 0.0366
P value summary *
Are the survival curves sig different? Yes

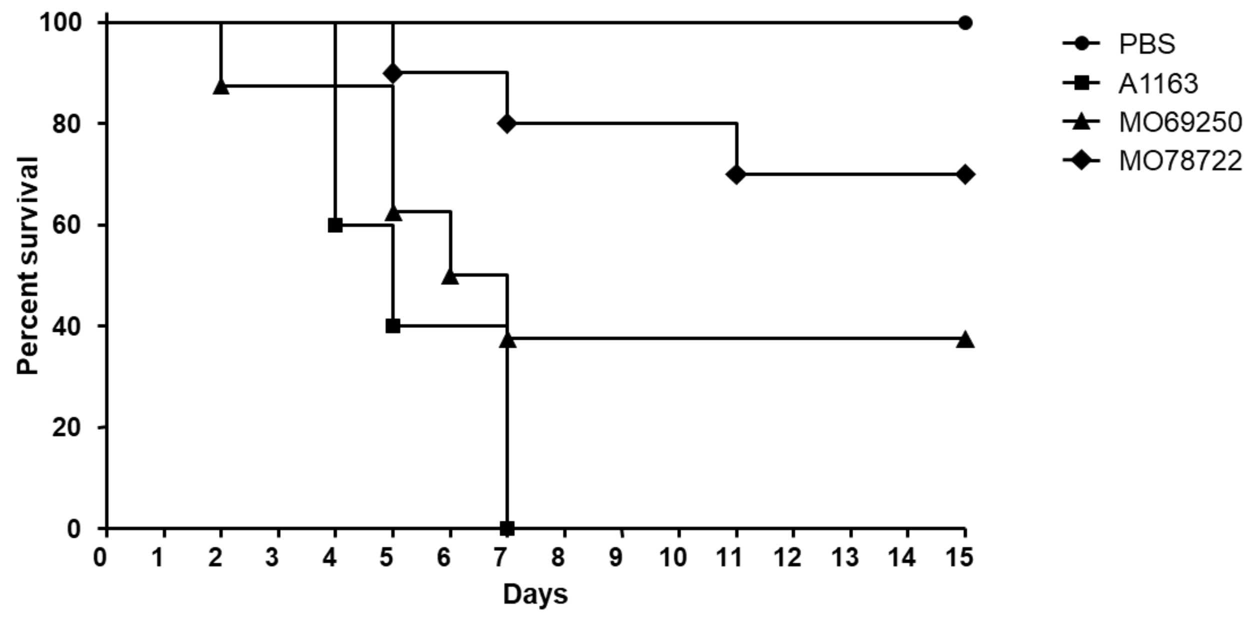

Median survival
A1163 5.000
MO69250 6.500

Log-rank (Mantel-Cox) Test
P value 0.2305
P value summary ns
Are the survival curves sig different? No

Gehan-Breslow-Wilcoxon Test
P value 0.2927
P value summary ns
Are the survival curves sig different? No

Median survival
A1163 5.000
MO78722 Undefined

Log-rank (Mantel-Cox) Test
P value 0.0015
P value summary **
Are the survival curves sig different? Yes

Gehan-Breslow-Wilcoxon Test
P value 0.0019
P value summary **
Are the survival curves sig different? Yes

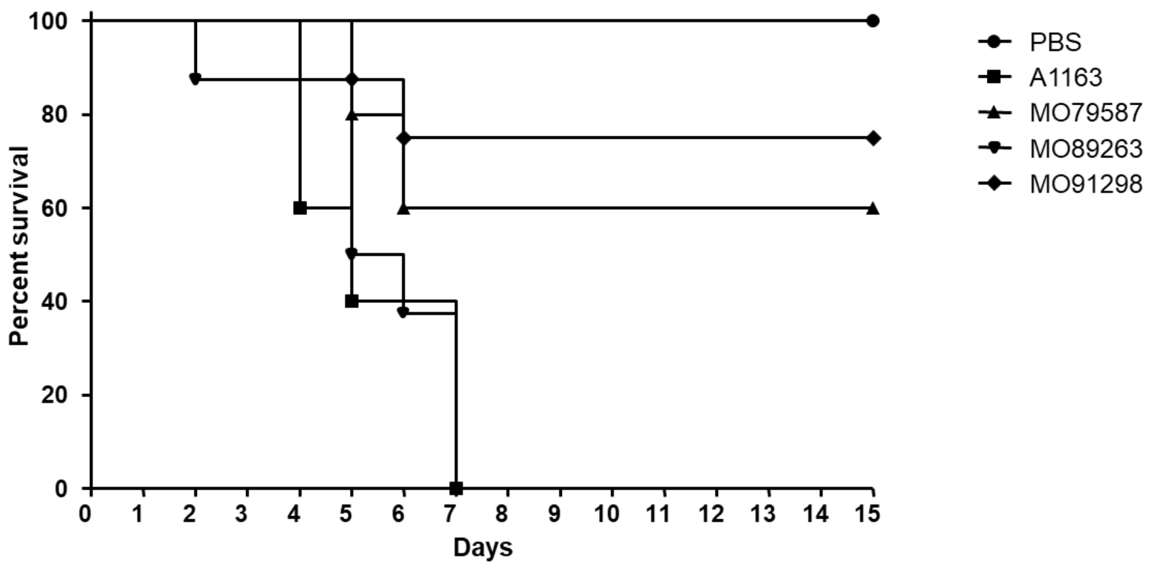

Median survival
A1163 5.000
MO79587 Undefined

Log-rank (Mantel-Cox) Test
P value 0.0234
P value summary *
Are the survival curves sig different? Yes

Gehan-Breslow-Wilcoxon Test
P value 0.0298
P value summary *
Are the survival curves sig different? Yes

Median survival
A1163 5.000
MO89263 5.500

Log-rank (Mantel-Cox) Test
P value 0.8370
P value summary ns
Are the survival curves sig different? No

Gehan-Breslow-Wilcoxon Test
P value 0.6994
P value summary ns
Are the survival curves sig different? No

Median survival
A1163 5.000
MO91298 Undefined

Log-rank (Mantel-Cox) Test
P value 0.0090
P value summary **
Are the survival curves sig different? Yes

Gehan-Breslow-Wilcoxon Test
P value 0.0117
P value summary *
Are the survival curves sig different? Yes

Figure S2. Strains Asfu1643, MO54056, MO68507, MO78722, MO79587 and MO91298 are less virulent than the reference strain A1163. Shown are survival curves of neutropenic mice infected via nasal inhalation with the respective strain. 10 mice were used for each strain and 5 mice for the PBS (phosphate buffered saline) negative control. Shown below each survival curve are the median survival of each strain and the P-values of two statistical tests (Mantel-Cox and Gehan-Breslow-Wilcoxon). All statistical tests are based on mouse survival compared to strain A1163.
